# Right Diaphragmatic Rupture: A Case Report of a Rare Surgical Trauma Emergency

**DOI:** 10.7759/cureus.42828

**Published:** 2023-08-01

**Authors:** Mohammad A Meaigel, Mohammed A Haider, Zisis Touloumis

**Affiliations:** 1 Trauma Surgery, King Saud University, Riyadh, SAU; 2 Trauma Surgery, King Saud Medical City, Riyadh, SAU

**Keywords:** blunt trauma, video-assisted thoracoscopic surgery (vats), blunt liver trauma, traumatic diaphragmatic rupture, traumatic diaphragmatic injury

## Abstract

Diaphragmatic injuries, particularly on the right side, are a rare yet challenging clinical scenario, especially when associated with other injuries. We present the case of a 38-year-old male patient who sustained a fall from a significant height, resulting in blunt abdominal trauma, chest injuries, right-side diaphragmatic injury, a grade 4 liver injury, and fractures of the right ribs, right femur, and pelvis. The patient also suffered a lung laceration with hemopneumothorax. The clinical team managed these injuries through a video-assisted thoracoscopy, laparotomy, and primary repair of the diaphragmatic rupture. The postoperative course was complicated by a low-output bile leak and infection of the orthopedic surgical wound, but these were effectively managed, and the patient showed a steady recovery. This case underscores the complexity of managing traumatic injuries that span multiple body regions and systems, requiring a coordinated, multidisciplinary approach. It also highlights the critical role of timely intervention and appropriate surgical strategies in the successful recovery of patients from complex traumas.

## Introduction

Right-side diaphragmatic injuries (RDIs) are not typically life-threatening but require operative repair and management [1]. Representing 1% to 5% of all traumatic injuries, traumatic diaphragmatic injuries (TDIs) are challenging to diagnose and associated with high mortality [2]. The incidence of RDI combined with organ injury is even lower, with liver injury present in 29% of all TDIs [3]. This case describes the challenging diagnosis, surgical management, and postoperative complications of a 38-year-old male patient who sustained an RDI and multiple organ injuries following a fall from a considerable height.

## Case presentation

**Figure 1:**
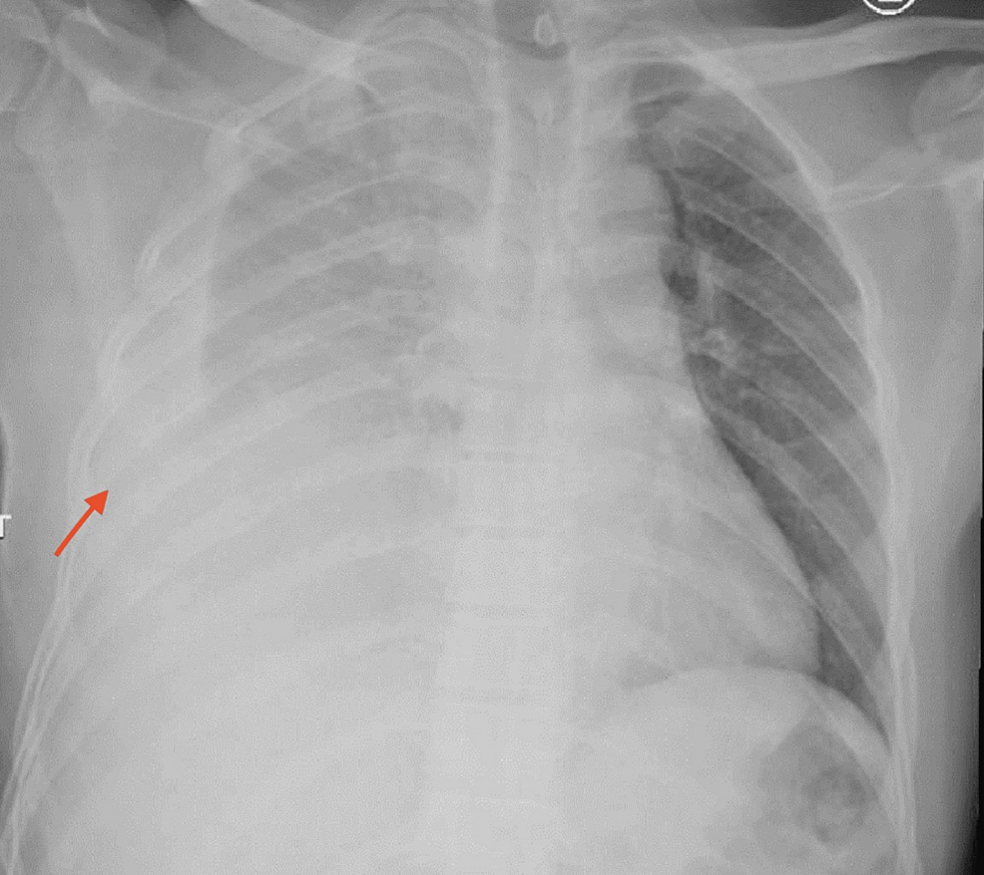
Initial chest X-ray showing right-sided hemothorax and elevated right diaphragm.

A 38-year-old male, who fell from a height of approximately 5 m, presented to our trauma center. He was hypoxic, hemodynamically unstable, and with a Glasgow Coma Scale of 8/15. After intubation and fluid resuscitation, a chest physical examination revealed decreased respiratory sounds on the right side. A chest X-ray showed a right hemothorax and an elevated right diaphragm (Figure 1), after which a right chest tube was inserted.

**Figure 2:**
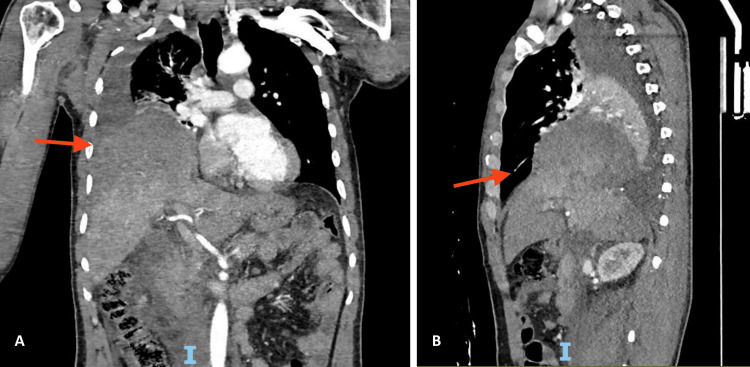
Coronal (A) and sagittal (B) computed tomography scan showing medium-to-large-sized hemothorax compressing the lung, right hemidiaphragm rupture with herniated liver into the right hemithorax, right hepatic lobe parenchymal disruption measuring approximately 12 × 5 cm, and large subcapsular and perihepatic hematoma representing grade 4 liver injury.

Following patient stabilization, he underwent computed tomography (CT) scans of the brain, chest, abdomen, pelvis, and skeletal survey X-rays. These revealed multiple hemorrhagic contusions, a small left occipital subdural hematoma, right hemothorax, right diaphragmatic rupture, liver lacerations (Figure 2), multiple pelvic fractures, and a right femur fracture.

Under the care of the trauma surgery department, the patient underwent video-assisted thoracoscopy (VATS) to clean the right chest cavity and inspect for possible lung parenchymal lacerations, followed by laparotomy. A 13-cm laceration was found on the posterior aspect of the right diaphragm and repaired with interrupted nylon sutures. After the liver was returned to the abdominal cavity, a right intercostal chest tube was adjusted, and a drain was inserted into the liver area.

During the patient’s postoperative hospital course, a low-output bile leak from the surgical drain was discovered on day three. On day five, the patient underwent endoscopic retrograde cholangiopancreatography (ERCP), which showed minimal intrahepatic right bile duct leakage, but no major ductal injury. Sphincterotomy was performed, and the drain output gradually decreased and stopped by day 25 after ERCP.

A postoperative chest collection was managed with postoperative intercostal tube (ICT) and pigtail drainage, with tubes removed on day 17 following the resolution of the collection. The patient’s pelvic and femur fractures were surgically managed on day five. He was extubated on postoperative day 12, at which time his orthopedic surgical wound showed signs of infection, leading to surgical debridement and plate removal.

## Discussion

Hemodynamic stabilization is the initial goal for all trauma patients. Life-threatening complications necessitate prompt intervention. In our patient’s case, his respiratory condition, hemothorax, and hypoxia required immediate management, achieved with intubation and ICT insertion.

Chest X-ray can suggest a diaphragmatic injury, but CT scans are the preferred diagnostic tool for assessing both diaphragm and liver injuries [4]. Our patient’s CT scan indicated a *hump and band* sign at the right diaphragm, aiding in accurate diagnosis and identifying the extent of the injury and complications [5].

Several surgical approaches can be adopted for patients with these injuries, as described in the literature. Laparotomy is often the optimal choice for fresh and combined injuries [6,7]. In our case, a combined approach via thoracoscopy and laparotomy was undertaken to clean the right chest cavity, check for potential lung parenchyma lacerations, and perform a definitive repair of the diaphragmatic and liver injuries.

Repair of the diaphragm is usually achievable through primary methods due to the general laxity of diaphragmatic tissue. If primary repair is not possible, alternative strategies such as mesh repair or adjacent local flap might be helpful [8].

Bile leak from liver injury is a known complication, and appropriate drainage is key for controlling sepsis in the lung or abdomen. However, major bile duct injury must be ruled out. ERCP can help diagnose and manage a major leak, with stenting if the injury is detected or simple sphincterotomy if no major biliary duct is injured [9].

## Conclusions

Diagnosis and management of RDI remain challenging, especially when associated with other organ injuries. In our experience, a combined approach involving VATS and laparotomy provides optimal management of both chest and abdominal cavities. This case underscores the importance of swift and comprehensive intervention for complex trauma cases, in which maintaining an interdisciplinary approach and leveraging advanced diagnostic and therapeutic techniques can significantly improve patient outcomes and survival rates.
